# Cetuximab as salvage monotherapy in chemotherapy-refractory metastatic colorectal cancer: A single-center report

**DOI:** 10.3892/ol.2013.1477

**Published:** 2013-07-19

**Authors:** EIJI MEKATA, YOSHIHIRO ENDO, HIROMICHI SONODA, TOMOHARU SHIMIZU, YUKI KAWAI, TOMOKO UMEDA, HISANORI SHIOMI, SHIGEYUKI NAKA, YOSHIHIRO KUBOTA, SATOSHI MURATA, HIROSHI YAMAMOTO, HAJIME ABE, TOHRU TANI

**Affiliations:** 1 Department of Surgery, Shiga University of Medical Science, Otsu, Shiga 520-2192, Japan; 2 Department of Clinical Nursing, Shiga University of Medical Science, Otsu, Shiga 520-2192, Japan

**Keywords:** cetuximab, epidermal gowth factor receptor, refractory, salvage treatment, metastatic colorectal cancer

## Abstract

In July 2008, cetuximab, a monoclonal antibody against epidermal growth factor receptor (EGFR), was approved in Japan for clinical use against chemotherapy-refractory metastatic colorectal cancer (mCRC). At Shiga University of Medical Science, between December 2007 and April 2012, a total of 24 EGFR-positive mCRC cases were administered immunohistochemistry with cetuximab as salvage monotherapy. The safety, side-effects and clinical efficacy of the treatment, including response rate, time to treatment failure, progression-free and overall survival, K-ras mutation status and impact on outcome, were investigated. The patient tumor growth control rate (TCR) was 38%, the mean time to progression (TTP) was 9.8 weeks [95% confidence interval (CI), 7.2–12.4] and the mean overall survival (OS) was 49.4 weeks (95% CI, 30.1–68.8). The most common adverse reactions reported were skin reactions, including acne (67%), hand-foot syndrome (16.7%) and paronychia (16.7%), followed by hypocalcemia (50%), hypomagnesemia (16%), stomatitis (20%) and gastrointestinal disorders (12%). The results of the present single-center study demonstrated that cetuximab monotherapy is beneficial for the treatment of chemotherapy-refractory patients with mCRC and that it has an acceptable level of safety and manageable side-effects.

## Introduction

Globally, colorectal cancer (CRC) is the second most common type of cancer diagnosed in females and the third most common in males, with >1.2 million new cases and 608,700 mortalities estimated to have occurred in 2008 (1). According to the Japanese cancer statistics of 2009, CRC was the third most common cause of cancer mortality in males, following lung and gastric cancer, and the first most common cause in females (2). While the cytotoxic agents, irinotecan, oxaliplatin and the fluoropyrimidines, and the monoclonal antibody, bevacizumab, have increased the median survival of patients with metastatic (m)CRC, with the exception of a minority of patients with resectable metastases, the disease remains incurable.

The monoclonal antibody, cetuximab, is directed against epidermal growth factor receptor (EGFR) and has exhibited beneficial activities in patients diagnosed with advanced CRC (3,4). Cetuximab was approved in Japan for clinical use in mCRC in July 2008. To date, only one study in Japan has analyzed the effects of cetuximab monotherapy on survival rate, progression-free survival and adverse effects (5). The present study aimed to investigate the outcome of using cetuximab as salvage monotherapy in 24 cases of patients with mCRC.

## Patients and methods

### Study design and eligibility criteria

In the current single-center study, cetuximab monotherapy was administered as salvage treatment. Eligibility requirements included histologically confirmed colorectal adenocarcinoma, surgically unresectable mCRC, advanced cancer refractory to fluoropyrimidine-, oxaliplatin- and irinotecan-based chemotherapies for which no other standard anticancer therapy was available and ≥1 unidimensionally measurable lesion. Patients were enrolled at Shiga University of Medical Science between December 2007 and April 2010 and had not received previous therapy directed against EGFR.

### Treatment

An intravenous loading dose of cetuximab (400 mg/m^2^ body surface area) was administered over a period of 120 min on day 1 of treatment, followed by an infusion of 250 mg/m^2^ body surface area administered over a period of 60 min once weekly.

### Assessments

Disease progression was documented by computed tomography or magnetic resonance imaging. Cetuximab therapy was continued until the disease progressed or until the patient was unable to tolerate the toxicity. Written informed consent was obtained from all participants.

### Statistical analysis

P<0.05 was considered to indicate a statistically significant difference. All statistical analyses were performed using SPSS version 19.0 for Windows (SPSS, Inc., Chicago, IL, USA).

## Results

### Patient population

The baseline characteristics of the 24 enrolled patients were as follows: 15 males and 9 females; median age, 69 years old (range, 36–88 years old); primary colon and rectal cancer, present in 54.2 and 45.8% of patients, respectively; and performance status (PS) 0, 1, 2 and 3, for 29.2, 29.2, 8.3 and 33.3%, respectively. KRAS mutation analyses performed by direct sequencing revealed KRAS mutations in codon 12/13 in the tumor tissue of 8/24 patients (33%). The most common metastatic sites were the lung (54.2%), liver (45.8%), lymph nodes (20.8%), peritoneum (12.5%) and bone (8.3%; Table I).

### Efficacy

The response rates to cetuximab are summarized in Table II. The median duration of follow up was 37 weeks. For all patients, the tumor growth control rate (TCR) was 38%, the mean time to progression (TTP) was 9.8 weeks [95% confidence interval (CI), 7.2–12.4] and the mean overall survival (OS) was 49.4 weeks (95% CI, 30.1–68.8).

In the wild-type KRAS subpopulation, 3 patients (19%) achieved a partial response (PR) and 6 patients (38%) achieved a PR or stable disease (SD). In the mutant KRAS subpopulation, 3 patients (38%) achieved a SD and no patients achieved a PR.

### Survival rates

**Figure 1:**
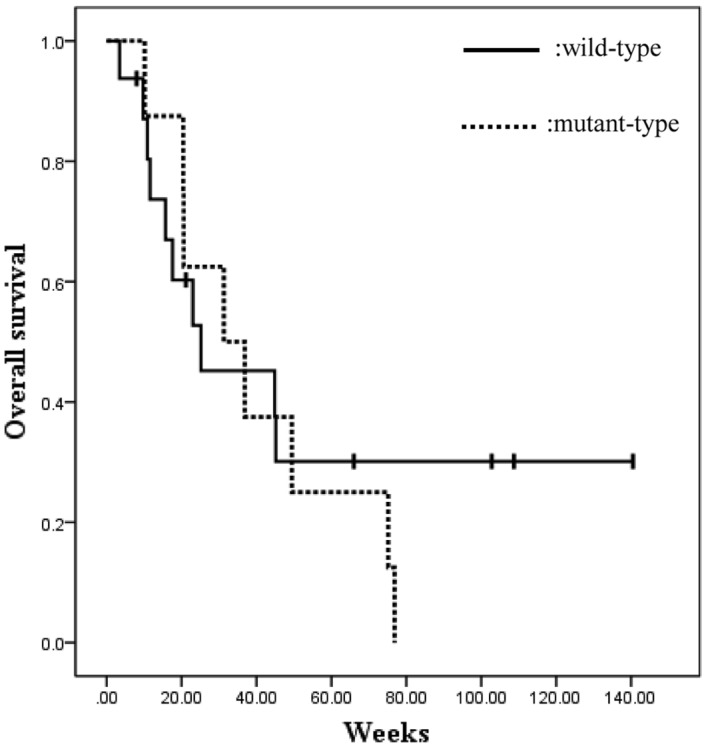
Kaplan-Meier survival plots for OS in advanced CRC treated with cetuximab monotherapy according to the KRAS type. OS, overall survival; CRC, colorectal cancer.

The mean OS times were 57.3 and 40.1 weeks for the wild-type KRAS and mutant KRAS groups, respectively. This difference was not statistically significant (P=0.584; log-rank test). However, 3 patients of the wild-type population survived >100 weeks (Fig. 1).

**Figure 2:**
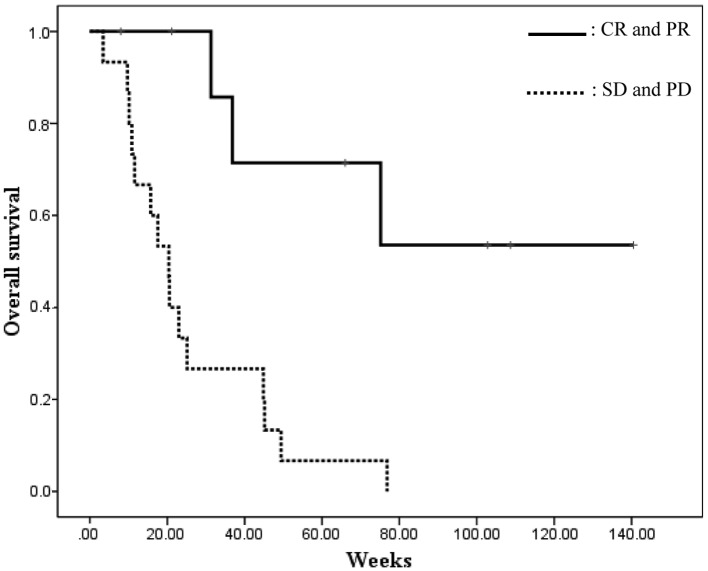
Kaplan-Meier survival plots for OS in advanced CRC treated with cetuximab monotherapy according to the evaluation of efficacy. OS, overall survival; CRC, colorectal cancer; CR, complete response; PR, partial response; SD, stable disease; PD, progressive disease.

**Figure 3:**
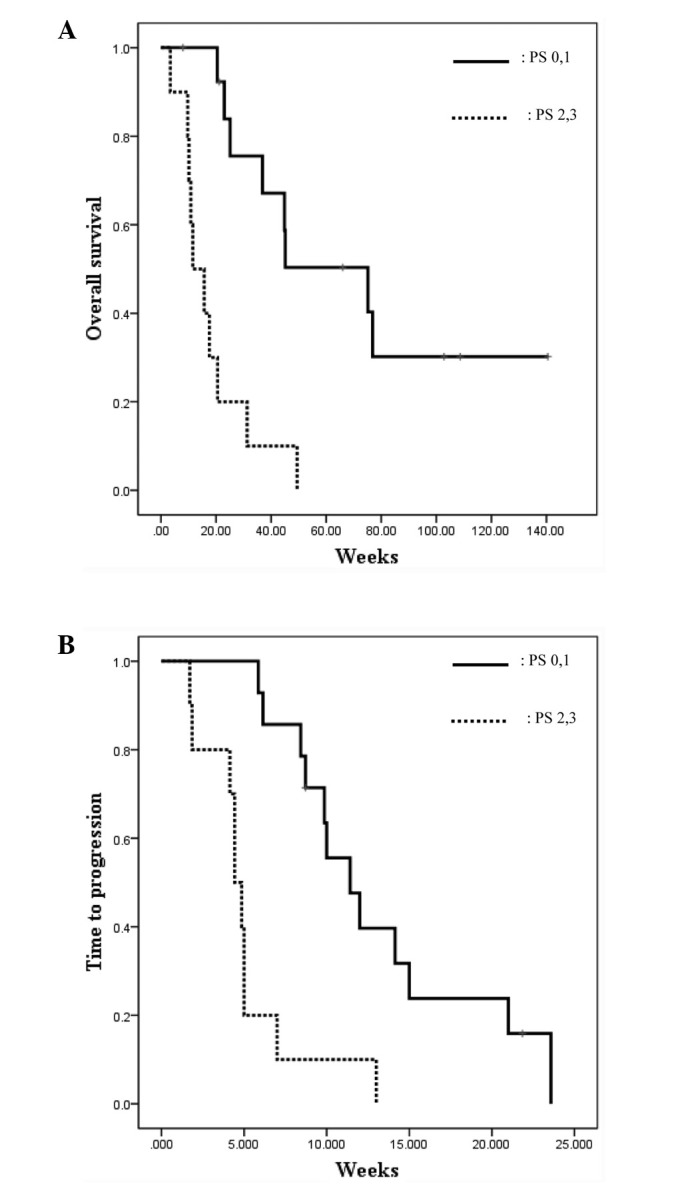
(A) Kaplan-Meier survival plots for OS and (B) TTP in advanced CRC treated with cetuximab monotherapy according to the performance status. OS, overall survival; TTP, time to progression; CRC, colorectal cancer; PS, performance status.

The mean OS of patients who exhibited a PR was higher compared with that of patients who did not (99.4 vs. 25.6 weeks; P=0.001; log-rank test; Fig. 2). The 1-year survival rate was >70% in the PR group, but <10% in the other survival groups. In addition, the median OS of patients who were PS0/1 was higher compared with that of patients who were not (74.0 vs. 18.0 weeks; P<0.001; log-rank test; Fig. 3A). PS was also the predictive factor of TTP (PS0/1 vs. PS2/3 groups, 13.2 vs. 5.1 weeks; P<0.001; log-rank test; Fig. 3B).

### Toxicity

With regard to toxicity the most common adverse effects reported were skin reactions, including acne (67%), hand-foot syndrome (16.7%) and paronychia (16.7%), followed by hypocalcemia (50%), hypomagnesemia (16%), stomatitis (20%) and gastrointestinal disorders (12%; Table III). One patient discontinued treatment due to grade 3 interstitial pneumonia. Additional grade 3 adverse effects were independent cases of stomatitis, acne and paronychia.

## Discussion

In recent years, the outcome of unresectable CRC has been improved by treatment with irinotecan, oxaliplatin and molecular target drugs. However, there is currently no consensus on the combination of drugs to use or the timing of administration. In addition, following standard chemotherapy the physical status of numerous mCRC patients worsens, but hope is maintained that subsequent treatments will result in a positive outcome. In these cases, treatments with fewer side-effects must be available, and cetuximab represents a clear candidate for this since cetuximab monotherapy is recommended for patients unable to tolerate combination chemotherapy (6). Therefore, in the present study, the efficacy and toxicity of cetuximab monotherapy for refractory mCRC was examined in Japanese individuals.

A PR was obtained in 3/24 patients (12.5%), a significant result for a population of patients who were previously only eligible for best supportive care prior to the introduction of anti-EGFR antibody. The overall disease control rate, including that for SD cases, was 37.5%. All 3 patients who achieved a PR were in the wild-type KRAS subpopulation, indicating that the anti-EGFR antibody is effective against KRAS wild-type disease. In addition, 2 of these patients showed such marked responses to treatment that the liver metastases were able to be removed.

In the mutant KRAS subpopulation, 2/8 patients (25%) were identified with SD and showed a significant decrease in tumor marker expression. Notably, these patients showed marked skin reactions. There are two hypotheses for the efficacy of the antibody in mutant cases. The first hypothesis is that the patients have a mutation at codon 13; cetuximab has been shown to be effective in specific patients with a mutation at codon 13 (7). Of the 2 patients with SD in the present study, 1 had a mutation at codon 12 and the other had a mutation at codon 13. It should also be noted that the two patients exhibited extremely strong skin reactions. The second hypothesis is the involvement of antibody-dependent cell-mediated cytotoxicity (ADCC). A previous *in vitro* study using a lung cancer cell line reported ADCC with cetuximab and suggested a correlation between EGFR expression levels and the magnitude of ADCC (8). In addition, Fc receptor polymorphisms have also been reported to be clinically relevant in mutated KRAS mCRC (9).

In the present study, monotherapy with the anti-EGFR antibody, cetuximab, demonstrated efficacy in the third-line or subsequent treatment of patients who exhibited resistance to anticancer agents. An evaluation of the effect of treatment demonstrated that an improved prognosis is expected in patients who achieved SD or PR and in those classified as PS0/1. Prognostic factors have been identified, including the occurrence of skin toxicity on therapy (10), the previous number of chemotherapy lines and early tumor shrinkage (11). Therefore, the results of the present study indicate that patients must be of good physical status to receive cetuximab treatment and that evaluations must be made during the early stages.

The current NCCN Guidelines (6) recommend anti-EGFR antibody therapy for patients with any number of prior therapy lines. However, anti-EGFR antibody therapy exerts early tumor shrinkage and thus is hypothesized to represent a suitable choice for the treatment of patients with advanced mCRC and a poor PS. Therefore, we recommend that cetuximab therapy is used for non-first-line treatment. Previous studies in other populations have identified that the therapy may improve the response rate and prolong survival when used as the second-line (12), third-line or subsequent (13) treatments. Future studies are required to determine the efficacy of the therapy in the Japanese population.

In conclusion, the results of the present study demonstrated the efficacy of cetuximab as a third-line treatment for Japanese patients with mCRC, however, additional analyses must be performed in the Japanese population to establish the optimal usage of the drug for the treatment of CRC.

## Figures and Tables

**Table I:** Patient characteristics.

Characteristics	Value
Patients, n	24
Gender, n (%)	
Male	15 (62.5)
Female	9 (37.5)
Age, years	
Median	69
Range	36–88
Performance status, n (%)	
0	7 (29.1)
1	7 (29.1)
2	2 (8.3)
3	8 (33.3)
Primary tumor site, n (%)	
Colon	13 (54)
Rectum	11 (46)
Sites of metastases, n (%)	
Liver	11 (45.8)
Lung	13 (54.2)
Lymph nodes	5 (20.8)
Peritoneum	3 (12.5)
Bone	2 (8.3)
Other	2 (8.3)
Prior chemotherapy regimens, n (%)	
2	0 (0.0)
3	11 (45.8)
>4	13 (54.2)

**Table II:** Efficacy of cetuximab monotherapy.

	KRAS status		
		
Best response	Wild-type (n=16)	Mutant (n=8)	Overall mean
CR, n	0	0	
PR, n	3	0	
SD, n	3	3	
PD, n	7	4	
NE, n	3	1	
Response, %	18.8	0	
Disease control, %	37.5	37.5	
Mean TTP, weeks	10.7	8.3	9.8
Mean OS, weeks	57.3	40.1	49.4

CR, complete response; PR, partial response; SD, stable disease; PD, progression of disease; TTP, time to progression; OS, overall survival; NE, not evaluated.

**Table III:** Adverse effects, (n=24).

	Grade, n (%)	
	
Effects	1 and 2	3 and 4
Non-hematological toxicities		
Gastrointestinal disorders	3 (12.5)	0 (0.0)
Diarrhea	2 (8.0)	0 (0.0)
Fatigue	0 (0.0)	1 (4.0)
Stomatitis	4 (16.7)	0 (0.0)
Hyperbilirubinemia	0 (0.0)	0 (0.0)
Hypomagnesemia	4 (16.7)	0 (0.0)
Hypocalcemia	12 (50)	0 (0.0)
Alopecia	0 (0.0)	0 (0.0)
Skin reaction		
Acne	16 (67)	1 (4.0)
Hand-foot syndrome	4 (16.7)	0 (0.0)
Dry skin	5 (20.8)	0 (0.0)
Paronychia	4 (16.7)	1 (4.0)
Peripheral neuropathy	2 (8.0)	0 (0.0)
Psychoneurotic disorder	1 (4.0)	0 (0.0)
Interstitial pneumonia	0 (0.0)	1 (4.0)
Ophthalmopathy	1 (4.0)	0 (0.0)
Infusion reaction	4 (16.7)	0 (0.0)

## Abbreviations

CR: complete response
CRC: colorectal cancer
ADCC: antibody-dependent cell-mediated cytotoxicity
EGFR: epidermal growth factor receptor
mCRC: metastatic colorectal cancer
OS: overall survival
PD: progessive disease
TTP: time to progression
PR: partial response
PS: performance status
SD: stable disease
